# Dysbiosis characteristics of gut microbiota in cerebral infarction patients

**DOI:** 10.1515/tnsci-2020-0117

**Published:** 2020-06-08

**Authors:** Hao Li, Xiaohui Zhang, Dengdeng Pan, Yongqiang Liu, Xuebing Yan, Yihan Tang, Mingyang Tao, Li Gong, Ting Zhang, Christian Rutan Woods, Yong Du, Renyuan Gao, Huanlong Qin

**Affiliations:** Department of General Surgery, Shanghai Tenth People's Hospital, Tongji University School of Medicine, Shanghai, 200072, China; Institute of Intestinal Diseases, Tongji University School of Medicine, Shanghai, 200072, China; Institute of Translational Medicine, Medical College, Yangzhou University, Yangzhou, 225001, China; Department of Neurology, Shanghai Tenth People's Hospital, Shanghai, 200072, China; Department of Biomedical Engineering, University of Houston, Houston, TX, 77204, United States of America; Diagnostic and Treatment Center for Refractory Diseases of Abdomen Surgery, Shanghai Tenth People's Hospital, Tongji University School of Medicine, Shanghai, 200072, China

**Keywords:** cerebral infarction, gut microbiota, dysbiosis, NIHSS, diagnosis

## Abstract

**Objective:**

The aim of this study is to investigate the dysbiosis characteristics of gut microbiota in patients with cerebral infarction (CI) and its clinical implications.

**Methods:**

Stool samples were collected from 79 CI patients and 98 healthy controls and subjected to 16S rRNA sequencing to identify stool microbes. Altered compositions and functions of gut microbiota in CI and its correlation with clinical features were investigated. Random forest and receiver operating characteristic analysis were used to develop a diagnostic model.

**Results:**

Microbiota diversity and structure between CI patients and healthy controls were overall similar. However, butyrate-producing bacteria (BPB) were significantly reduced in CI patients, while lactic acid bacteria (LAB) were increased. Genetically, BPB-related functional genes were reduced in CI patients, whereas LAB-related genes were enhanced. The interbacterial correlations among BPB in CI patients were less prominent than those in healthy controls. Clinically, BPB was negatively associated with the National Institutes of Health Stroke Scale (NIHSS), while LAB was positively correlated with NIHSS. Both BPB and LAB played leading roles in the diagnostic model based on 47 bacteria.

**Conclusions:**

The abundance and functions of BPB in CI patients were significantly decreased, while LAB were increased. Both BPB and LAB displayed promising potential in the assessment and diagnosis of CI.

## 1 Introduction

Cerebral infarction (CI) refers to severe brain tissue damage due to prolonged ischemia usually resulting from an embolus or thrombus [1]. It is one of the leading causes of mortality worldwide, accounting for 5.2% of global deaths. CI is associated with the highest disability rate in China with over 2 million new cases annually resulting in sequelae such as hemiplegia and aphasia [2]. Common risk factors of CI include hypertension, diabetes, smoking, and obesity [3]. Despite considerable research efforts, the pathogenic factors of CI have not been fully elucidated. Therefore, it is crucial to identify the underlying mechanisms of CI to assist in its prevention and successful therapeutic intervention.

Previous studies have implicated that gut microbiota dysbiosis plays a role in the pathophysiology of CI through multiple mechanisms. The gut microbial metabolite trimethylamine *N*-oxide (TMAO) was shown to be independently associated with the incident risk of thrombotic events such as stroke [4]. Alternatively, researchers demonstrated that gut microbiota plays a role in the outcome of ischemic stroke in an antibiotic-induced mouse model of intestinal dysbiosis [5]. Despite the emergence of an association between gut microbiota dysbiosis and CI, the specific dysbiosis-related bacteria still need to be elucidated.

Gut microbiota dysbiosis has been further characterized in cardiovascular diseases compared to CI. Considering the significant link between cardiovascular diseases and stroke, we hypothesized that there would be similar alterations in the gut microbiota. Butyrate-producing bacteria (BPB) are a type of bacteria characterized by the production of butyric acid, which plays an important role in providing energy to the intestinal epithelium and regulating the immune system [6]. Studies have shown increased BPB to have a protective effect against cardiovascular disease [7]. BPB at the genus level include *Faecalibacterium* and *Subdoligranulum* of the family Ruminococcaceae, *Roseburia* and *Lachnoclostridium* of the family Lachnospiraceae, *Butyricicoccus* and *Clostridium* of the family Clostridiaceae, *Eubacterium* of the family *Eubacteriaceae*, and so on [8,9]. Among these families, the Ruminococcaceae and Lachnospiraceae bacteria were described as spore formers [10]. Considering the high abundance of these two bacteria in gut microbiota [11], any changes observed in sporulation could be considered a reflection of the functional changes of BPB in CI. In addition, lactic acid bacteria (LAB) such as *Lactobacillus* and *Lactococcus* have also been found to be favorably involved in cardiovascular diseases by lowering cholesterol [12]. LAB produce the lactic acid through one of the functional genes phosphotransferase system (PTS) [13], which also parallelly represents changes in the LAB function. These favorable associations of BPB and LAB with cardiovascular disease make them intriguing targets to investigate when characterizing gut microbiota dysbiosis in CI.

In this study, we set out to investigate the changes in gut microbiota abundance and diversity between CI patients and healthy controls (HCs) and to observe whether these variations are correlated with clinical parameters. To do this, we analyzed stool samples from 79 CI patients and 98 HCs by 16S rRNA sequencing. Given the potential implication of BPB and LAB in CI, these two groups of bacteria were particularly investigated. This information was used to observe whether there was a correlation between gut microbiota and clinical parameters, especially the National Institutes of Health Stroke Scale (NIHSS) score. NIHSS is a tool for the quantitative measurement of stroke-related neurological deficits [14], with higher scores indicating more severe disease. Based on the significance of altered gut microbiota in CI, a novel diagnostic model was proposed in our study.

## 2 Methods

### 2.1 Ethics statement and patients

All CI patients underwent computed tomography and were diagnosed by a neurologist. Patient data included NIHSS scores, blood pressure measurements, demographics, and medical histories. Initial exclusion criteria included cancer, infection, history of intestinal disease, or exposure to antibiotics or probiotics within 1 month before sample collection. Individuals with neurological disorders, neuropsychiatric diseases, or a history of craniocerebral surgery were further excluded from the study. Healthy volunteers were recruited in the community, and the detailed information was recorded using questionnaires.


**Ethical approval:** The research related to human use has been complied with all the relevant national regulations, institutional policies, and in accordance the tenets of the Helsinki Declaration and has been approved by the Ethics Committee of Shanghai Tenth People's Hospital (China).
**Informed consent:** Informed consent has been obtained from all individuals included in this study.

### 2.2 Sample collection

All subjects were provided with collection boxes and disposable sterile forceps for stool sample collection. Collection date and patient name were recorded on each collection box. All samples were immediately placed on ice for transport to the laboratory within 1 h of collection. On arrival to the laboratory, all samples were immediately aliquoted and stored in a −80℃ freezer.

### 2.3 DNA extraction

DNA extractions were performed using QIAamp DNA Stool Mini Kit (Qiagen, USA) according to the manufacturer’s instructions. DNA extraction was performed within 15 days of sample collection, and the extracted DNA was stored at −20℃.

### 2.4 Pyrosequencing and bioinformatics analysis

Sequencing and bioinformatics analysis were carried out as previously described [15]. Briefly, the 16S ribosomal RNA gene was amplified in triplicate using the quantitative real-time polymerase chain reaction. The purified products were pyrosequenced using the Miseq system (Illumina, San Diego, California). The data obtained by sequencing were optimized for operational taxonomic units (OTUs) clustering and used for different analysis, including the alpha and beta diversity analyses, the principal coordinates analysis (PCoA), and the linear discriminant analysis effect size (LEfSe) to characterize the differences between CI and HCs. The function prediction analysis was performed using the Phylogenetic Investigation of Communities by Reconstruction of Unobserved States (PICRUSt) software based on KEGG (Kyoto Encyclopedia of Genes and Genomes) databases. Correlations between gut microbiota and clinical parameters were evaluated by the Spearman and Pearson test. Random forest and receiver operating characteristic (ROC) curve were used in the development of a diagnostic model.

### 2.5 Statistical analysis

The quantitative data were statistically analyzed by Student's *t*-test or Mann–Whitney *U* test (GraphPad Prism 7.00, USA). The qualitative data were compared by the chi-square test (SPSS 24.0, IBM). The Bonferroni method was used to correct the false discovery rate (FDR) of the microbiota correlation analysis. Two-tailed *P* value <0.05 was considered statistically significant.

## 3 Results

### 3.1 Characteristics of enrolled subjects

A total of 79 CI patients and 98 HCs were included in this study (Figure S1). There was no significant difference observed between these two groups regarding proportions of gender, smoking, comorbidities, average age, or body mass index (BMI). The average NIHSS score of CI was 1.75 ± 2.75 (Table 1).

**Table 1:** Baseline characteristics of study population with CI and HCs

	Cerebral infarction(*n* = 79)	Healthy control(*n* = 98)	*P* value
Age (year)	66.61 ± 12.07	64.01 ± 10.44	0.126
Female (%)	29 (36.7)	41 (41.8)	0.488
BMI (kg m^−2^)	24.22 ± 2.34	23.83 ± 2.11	0.246
NIHSS score	1.75 ± 2.75	–	–
Smoking (%)	12 (15.2)	10 (10.2)	0.364
Diabetes (%)	28 (35.4)	22 (22.4)	0.066
Hypertension (%)	56 (70.9)	58 (59.2)	0.117

Data expressed as *n* (%) or mean ± SD. *P* values calculated by using Student *t* test or Chi-squared test. BMI: body mass indexes, NIHSS: National Institutes of Health Stroke Scale.

### 3.2 Similar microbiota diversity and structure between CI patients and HCs

**Figure 1:**
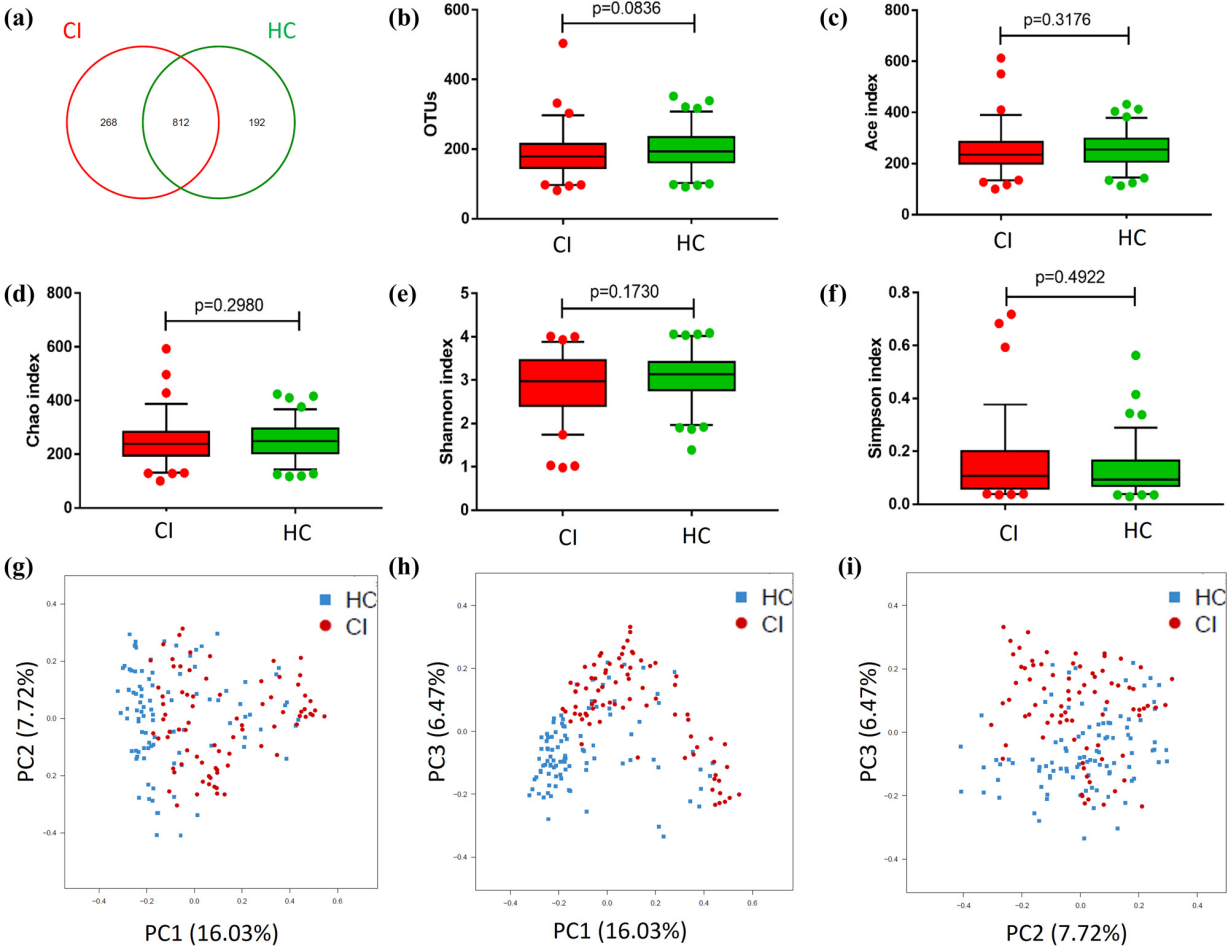
Similar microbiota diversity and structures between CI patients and HCs. (a) Venn diagram showing the overlapping OTU data of the two groups. (b–f) OTUs, richness (Ace and Chao index), and diversity (Shannon and Simpson index) between the CI patients and HCs were not significantly different. (g–i) Two groups could not be separated based on the relative abundance of genera by PCoA, indicating a similar microbiota structure.

By using the 16S rRNA gene sequencing analysis, we identified a total of 1,272 OTUs. Of the detected OTUs, 268 and 192 were found to be specific to the CI and HCs groups, respectively (Figure 1a). The number of OTUs (179 vs 192.5, *P* = 0.0836) (Figure 1b) and richness, represented by the abundance-based coverage estimator (ACE) (235 vs 254, *P* = 0.3176) and Chao (239 vs 248, *P* = 0.2980) indexes, were not significantly different between CI patients and HCs (Figure 1c and d). The diversity of gut microbiota represented by Shannon index and Simpson index was lower in CI patients than that of HC, but no significant difference was identified (2.97 vs 3.14, *P* = 0.1730) (Figure 1e and f). PCoA demonstrated that two groups could not be separated based on the relative abundance of genera (Figure 1g–i), indicating that these two groups had similar microbiota structures.

### 3.3 Altered abundances of BPB and LAB in CI patients

**Figure 2:**
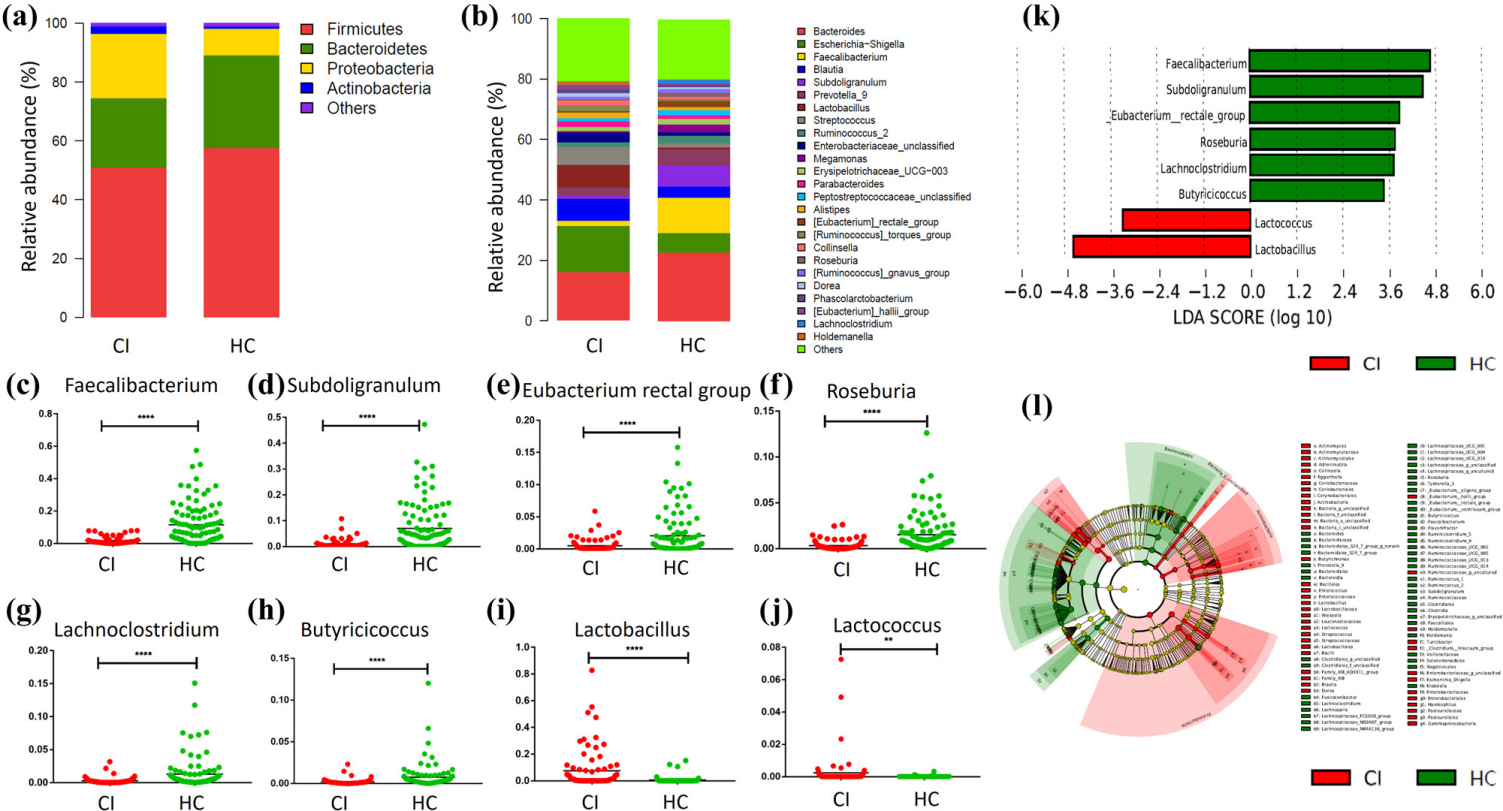
Altered abundances of BPB and LAB in CI patients. The relative taxa abundance at the phylum (a) and genus (b) level between CI group and HC. (c–h) The six BPB including *Faecalibacterium*, *Subdoligranulum*, *Eubacterium rectal group*, *Roseburia*, *Lachnoclostridium*, and *Butyricicoccus* decreased significantly in the CI group compared with HC. (i and j) The two LAB including *Lactobacillus* and *Lactococcus* increased significantly in the CI group. (k) The LEfSe analysis evaluated by the LDA score indicated that all BPB and LAB could characterize the differences between CI patients and HC. (l) Cladogram showing the structures and abundance of the gut microbiota in the CI and HC.

At the phylum level, the relative abundance of Firmicutes (50.93% vs 57.55%, FDR = 0.049) and Bacteroidetes (23.53% vs 31.64%, FDR = 0.007) decreased, while Proteobacteria (21.99% vs 9.03%, FDR = 1.09 × 10^−6^) and Actinobacteria (2.45% vs 0.75%, FDR = 6.26 × 10^−9^) increased in the CI patients compared with HCs (Figure 2a).

At the genus level, several genera were significantly different in the CI group in comparison with the HCs (Figure 2b and Figure S2), particularly the BPB and LAB. The abundances of six BPB, including *Faecalibacterium* (0.94% vs 7.32%, *P* < 0.0001), *Subdoligranulum* (0.35% vs 3.44%, *P* < 0.0001), *Eubacterium rectal* group (0.13% vs 0.47%, *P* < 0.0001), *Roseburia* (0.12% vs 0.80%, *P* < 0.0001), *Lachnoclostridium* (0.11% vs 0.47%, *P* < 0.0001), and *Butyricicoccus* (0.10% vs 0.32%, *P* < 0.0001), decreased significantly in the CI patients (Figure 2c–h). On the contrary, the abundance of two LAB, including *Lactobacillus* (0.95% vs 0.01%, *P* < 0.0001) and *Lactococcus* (0.0056% vs 0%, *P* < 0.01), were significantly increased in the CI patient sample (Figure 2i and j). Furthermore, the LEfSe analysis evaluated by the linear discriminant analysis (LDA) score demonstrated that these six BPB were key bacteria in the HC group, whereas the two LAB were major bacteria in CI patients (LDA > 2.4, *P* < 0.05, Figure 2k). These results indicate that each of these BPB and LAB could assist in characterizing the differences between CI patients and HCs. A cladogram was constructed to visually represent these results. The green color indicates branches of the phylogenetic tree, which more significantly represent the HCs, while the red color indicates those that significantly represent CI patients (Figure 2l). Other microbiota found to be significantly different include *Escherichia* and *Shigella* (15.31% vs 6.44%, FDR = 1.14 × 10^−4^), *Streptococcus* (5.97% vs 1.43%, FDR = 0.002), *Collinsella* (1.73% vs 0.53%, FDR = 0.003), and *Dorea* (1.30% vs 0.62%, FDR = 0.005) (Figure 2b and Figure S2).

### 3.4 Altered BPB and LAB-related functional genes in CI patients

**Figure 3:**
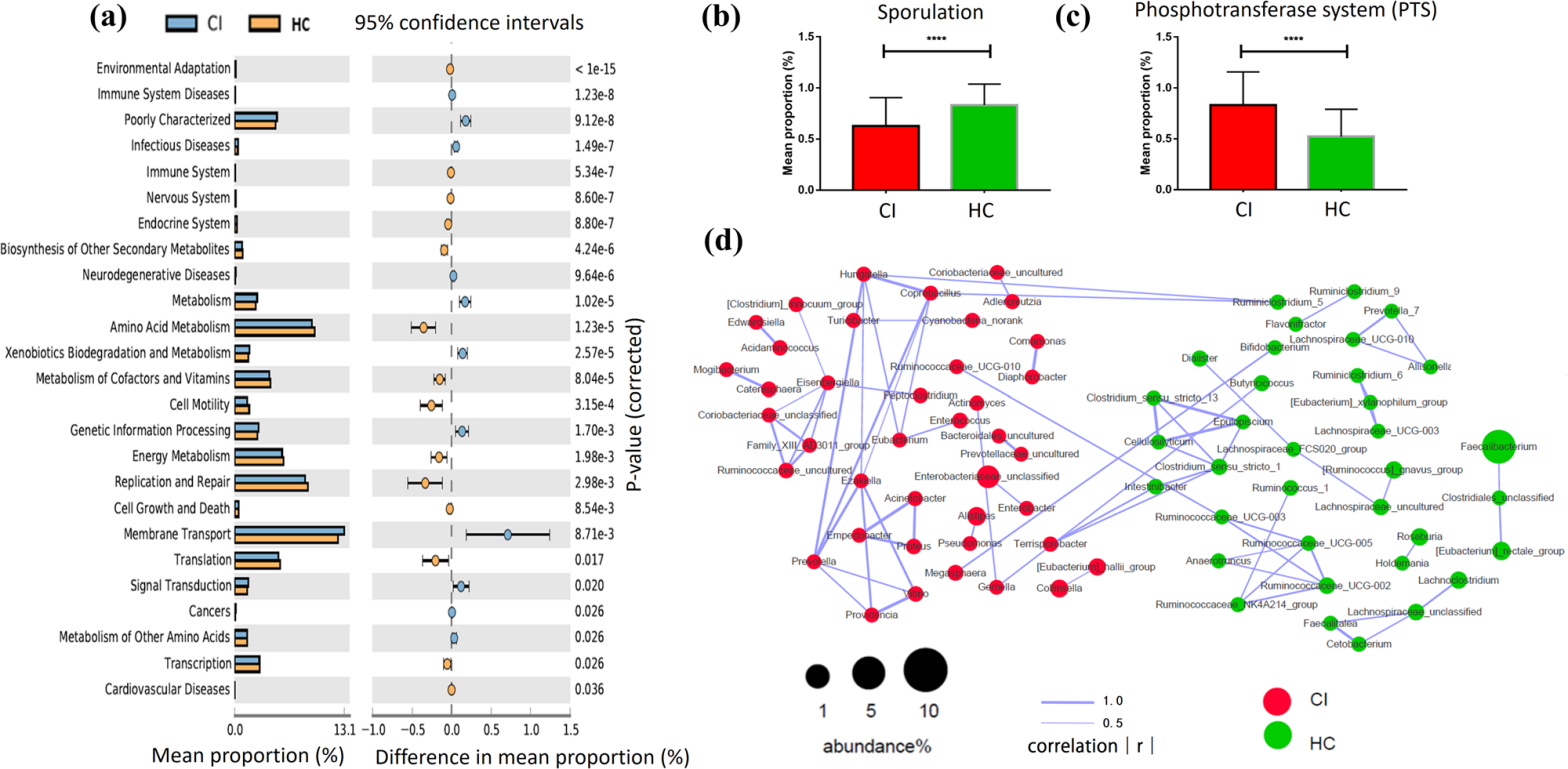
Altered BPB and LAB-related functional genes in CI patients and less interbacterial correlations among BPB in CI patients. (a) The difference between the functional genes was evaluated between CI patients and HC based on the KEGG of level 2. (b) BPB-related sporulation functional genes were significantly decreased in CI patients at the KEGG of level 3. (c) LAB-related PTS genes were increased in CI patients at the KEGG of level 3. (d) The microbial network showed that the interbacterial relationships among BPB in CI patients were less prominent than that among HC group. No correlations among the BPB including *Clostridium_innocuum_group*, *Ruminococcaceae_UCG-010*, *Eubacterium*, and *Ruminococcaceae_uncultured* were found in the CI group. However, many correlations among BPB families, including *Ruminococcaceae*, *Lachnospiraceae*, and *Clostridiaceae*, were identified in the HC group.

To reveal the functional alterations associated with taxonomic changes, we compared the predicted functional differences in the gut community between CI and HC groups. Based on KEGG of level 2, the cellular process module decreased, while the human disease-associated module increased in the CI group. Genes corresponding to infectious diseases, metabolism, genetic information processing, membrane transport, and signal transduction were all highly abundant in the CI group. Alternatively, genes related to amino acid metabolism, cell motility, replication and repair, and translation were enriched in the HC group (Figure 3a). Furthermore, BPB-related sporulation functional genes were significantly decreased (*P* < 0.0001) in CI patients (Figure 3b), and LAB-related PTS genes were significantly increased (*P* < 0.0001) in CI patients at KEGG of level 3 (Figure 3c).

### 3.5 Decreased interbacterial correlations among BPB in CI patients

A microbial network based on bacteria with abundance over 1% (|*r*| > 0.3, *P* < 0.05) showed interbacterial correlations in the CI group to be stronger than that in the HCs. *Eisenbergiella*, *Prevotella*, *Ezakiella*, *Coprobacillus*, and *Hungatella* were each associated with at least five other bacteria in the CI group, while only *Clostridium_sensu_stricto_1* was associated with five other bacteria in the HC group (Figure 3d). However, the interbacterial relationships among BPB in CI patients were less prominent than that among HC group. No correlations were discovered among the BPB families *Clostridium_innocuum_group*, *Ruminococcaceae_UCG-010*, *Eubacterium*, or *Ruminococcaceae_uncultured* in the CI group. Conversely, many correlations were identified in the HC group among BPB families, including *Ruminococcaceae*, *Lachnospiraceae*, and *Clostridiaceae* (Figure 3d).

### 3.6 The clinical implications of BPB and LAB in CI severity assessment and diagnosis

**Figure 4:**
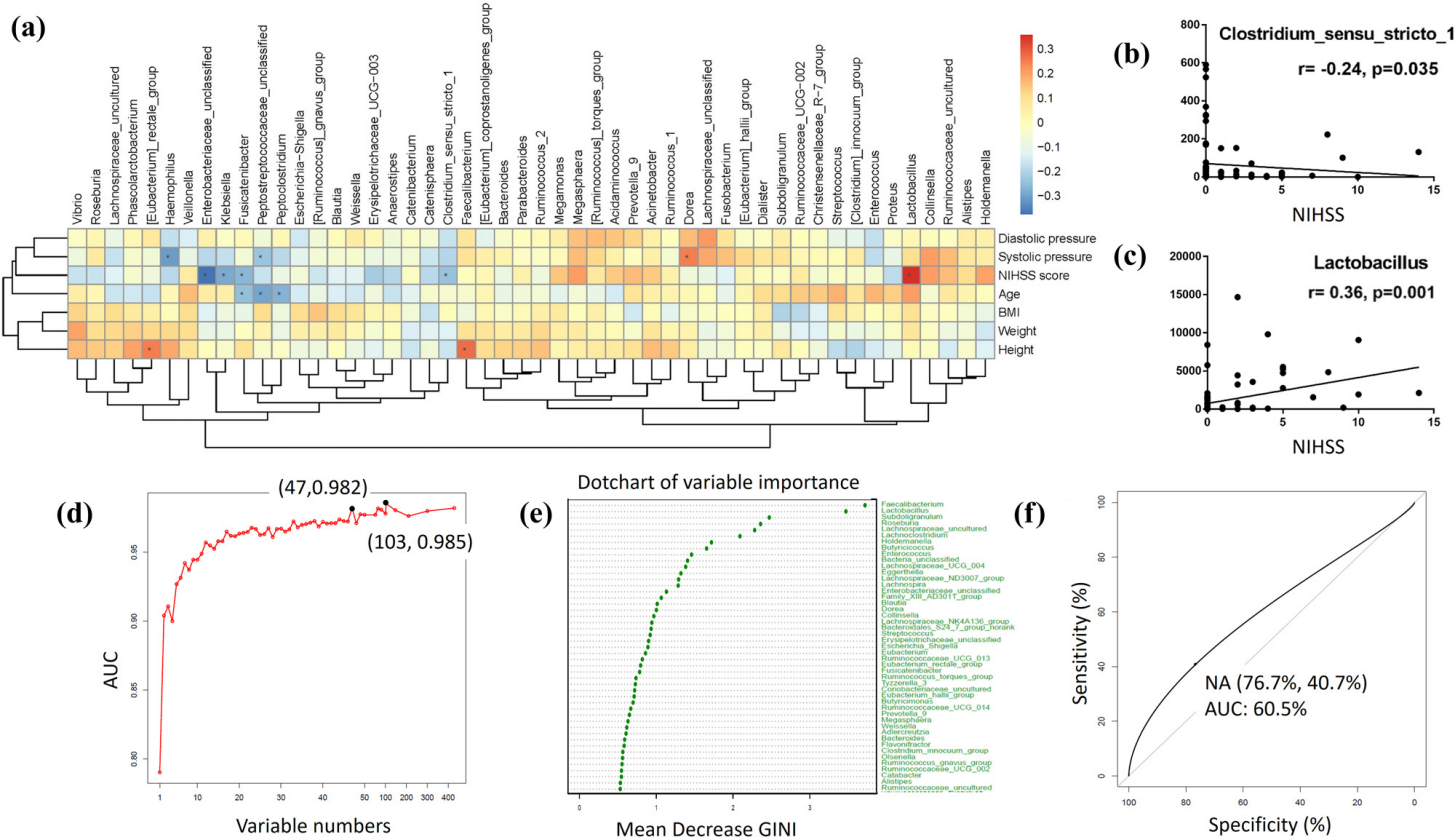
The clinical implications of BPB and LAB in CI severity assessment and diagnosis. (a) Correlation analysis was conducted between gut microbiota and clinical parameters. *P < 0.05. (b and c) NIHSS score was negatively associated with the BPB *Clostridium_sensu_stricto_1* and positively associated with the LAB *Lactobacillus*. (d) The optimal variable number (47 variables) was decided by the random forest method. (e) The six BPB and the LAB *Lactobacillus* played leading roles among the 47 bacteria based on the mean decreased Gini index. (f) The area under the curve of ROC was 0.605.

The correlation analysis demonstrated that NIHSS score was negatively associated with the BPB *Clostridium_sensu_stricto_1* abundance (*r* = −0.24, *P* = 0.035) (Figure 4a and b) and positively associated with the LAB *Lactobacillus* abundance (*r* = 0.36, *P* = 0.001) in CI patients (Figure 4a and c). NIHSS score was also negatively associated with *Enterobacteriaceae_unclassified* (*r* = −0.38, *P* = 0.001), *Klebsiella* (*r* = −0.29, *P* = 0.011), and *Fusicatenibacter* (*r* = −0.29, *P* = 0.021). In addition, age was negatively associated with *Peptostreptococcaceae_unclassified* (*r* = −0.29, *P* = 0.009), *Peptoclostridium* (*r* = −0.27, *P* = 0.018), and *Fusicatenibacter* (*r* = −0.25, *P* = 0.027). Height was positively associated with *Faecalibacterium* (*r* = 0.27, *P* = 0.015) and *Eubacterium_rectale_group* (*r* = 0.25, *P* = 0.029). Systolic pressure was negatively associated with *Haemophilus* (*r* = −0.31, *P* = 0.006) and *Peptostreptococcaceae_unclassified* (*r* = −0.24, *P* = 0.036) and was positively associated with *Dorea* (*r* = 0.25, *P* = 0.029) (Figure 4a).

Random forest was used to decide the optimal variable number in our diagnostic model. The maximum area under the curve (AUC) (0.985) was obtained using 103 variables, but a panel of 47 variables was more feasible to construct the diagnostic model without yielding much discriminating potential (AUC = 0.982) (Figure 4d). These were chosen by ranking bacteria according to their mean decrease in Gini Index and selecting the top 47 (Figure 4e). Among these bacteria, the BPBs *Faecalibacterium* and *Subdoligranulum* along with the LAB *Lactobacillus* were found to be the three most significant in the diagnostic model. By using this panel, we obtained an AUC of 0.605 (95% confidence interval: 0.5185–0.6916) and accuracy of 60.6% after 10-fold cross-validation for the diagnosis of CI. The sensitivity and specificity were 40.7%, and 76.7%, respectively (Figure 4f).

## 4 Discussion

The role gut microbiota dysbiosis plays in CI remains a novel area of research. We found the gut microbiota compositions of CI patients and HCs to be similar overall; however, there were significant differences observed between certain bacterial population. Of these, BPB abundances were significantly reduced in CI patients, and there was a negative correlation between BPB and NIHSS. The BPB-related functions were attenuated, and the interbacterial correlations among BPB were less prominent in CI patients. Conversely, the abundances of LAB in CI patients were increased, and LAB were positively correlated with the disease severity. Both BPB and LAB contributed significantly in the construction of the CI diagnostic model. In addition, our study revealed that some pathogenic bacteria, such as *Escherichia* and *Shigella*, were significantly increased in patients with CI.

Other recent studies have also investigated the dysbiosis characteristics associated with CI. Consistent with our findings, Li et al. reported no significant difference in the alpha diversity and microbiota structures between CI patients and HCs. However, they reported that the abundances of short-chain fatty acid-producing bacteria (including BPB) were significantly higher in CI patients compared to HCs, which contradicts our observations [16]. This discrepancy might be due to sample size difference, different regional dietary habits of included patients, or the differences of BPB at the genus level. Alternatively, another recent study published by Zeng et al. was in agreeance with our findings regarding BPB. While our study compared CI patients with HCs, Zeng et al.'s study focused on the difference in gut microbiota dysbiosis between different CI risk groups. They reported decreases in BPB abundances and enriched LAB in the high-risk CI group [17], further confirming our results in populations with predisease status of CI. Zeng et al. went on to validate their results by using gas chromatography as a secondary technique to measure the level of butyrate produced by BPB in samples. Despite the similarities in our BPB findings, there were conflicting results when it came to LAB. They found the LAB *Bifidobacterium* to be significantly enriched in CI patient samples, while we did not observe a significant difference in these bacteria. This discrepancy may be due to their classification of different disease statuses or different regional dietary habits of included patients. Overall, the small sample sizes or geographical differences of sample population may contribute to the disparities of these three studies.

Previous studies have demonstrated a link between reduced BPB abundance and metabolic diseases, such as obesity and diabetes, which are risk factors of CI [18]. *In vivo* experiments suggested that butyrate could prevent the development of insulin resistance and obesity [19,20]. In our study, the BPBs *Subdoligranulum* (*r* = −0.20, *P* = 0.07) and *Ruminococcaceae_UCG-002* (*r* = −0.19, *P* = 0.09) were found to have the highest negative correlation with BMI although there was no statistical difference. We interpret this as an indication that the decrease of BPB was associated with an increase of BMI in CI patients.

In addition to the link between BPB and metabolic diseases, other animal studies have confirmed that butyrate has a protective effect in CI. It has been reported that sodium butyrate could stimulate neurogenesis through mitigating the microglia-mediated neuroinflammation effect in the ischemic brain [21,22,23]. Given the negative correlation between NIHSS score and the BPB *Clostridium_sensu_stricto_1* (*r* = −0.24, *P* = 0.035), we speculate that the reduced BPB caused a significant butyrate decrease in CI patients. This decrease could result in impaired inflammation inhibition of the infarction and thus relate with the severity of CI. More research is needed to clarify the mechanism of BPB reduction in CI and the subsequent effects as there are currently no published studies on this subject.

We originally hypothesized about the potential role of BPB reduction in CI based on the existing literature. The increased red meat intake and the reduced fiber intake lead to a reduction of BPB abundance [24], which causes an imbalance of gut microbiota. The reduction of BPB abundance results in the decreased production of butyrate, including the histone deacetylase inhibitor sodium butyrate. Studies have shown that sodium butyrate can significantly diminish the size of CI infarct and limit the brain damage [25]. In addition, butyrate could improve atherosclerosis through the upregulation of ABCA1 expression and cholesterol efflux in macrophages through the Sp1 pathway [26]. Therefore, it is reasonable to conclude that a reduction of BPB enhances the chance of CI occurrence (Figure S3).

LAB including *Lactobacillus* and *Lactococcus* were significantly increased in CI patients, which seemingly contradicted the beneficial roles of LAB seen in cardiovascular diseases [27,28]. Interestingly, studies have confirmed that lactate is mainly fermented to butyrate by BPB [29,30], which could reasonably explain the significant increase of LAB in CI patients. Considering the butyrate is good for reducing inflammation and protecting brain, as its level decreases, LAB abundance compensatorily increases to produce more lactate for fermentation to butyrate (Figure S3). In addition, by the co-culture of *Faecalibacterium prausnitzii* (BPB) and *Bifidobacterium adolescentis* (LAB) *in vitro*, David et al. found that *Bifidobacterium adolescentis* could promote *F. prausnitzii* to generate more butyrate by providing more acetate [31]. This indicates that the interaction between the two bacteria is independent of CI. Further elucidation of the specific relationships between BPB and LAB in CI patients requires further study.

In addition to the BPB and LAB findings, our results showed Actinobacteria and Proteobacteria abundances to be significantly increased in CI patients (Figure 2a). In a previous study, Romano et al. found that the trimethylamine (TMA)-producing microbiota were mainly characterized by the presence of the choline-TMA lyase system (CutC/D) [32], which includes Actinobacteria, Proteobacteria, and some other bacteria [33]. This suggests that there is increased TMA production in the gut of patients with CI. This excessive TMA is further oxidized to TMAO in the liver by flavin-containing monooxygenases [34]. TMAO activates mitogen-activated protein kinase (MAPK) and NF-κB signals in vascular smooth muscle cells and endothelial cells, leading to inflammatory gene expression and endothelial cell leukocyte adhesion. In addition, TMAO increases the expression of the scavenger receptors CD36 and SR-A1 *in vivo*, which induce macrophages to take up more modified LDL to form foam cells. TMAO also increases the release of endoplasmic reticulum calcium from platelet cells, leading to platelet aggregation and thrombosis [35,36,37]. All these features of TMAO increase the risk of CI (Figure S3).

There are some limitations of this study. First, the area under the curve of the diagnostic model was not high (0.605). However, compared with serological parameters, the model still showed a prospective follow-up application due to its noninvasive characteristics. The low sensitivity (40.7%) of the model indicates that it is prone to false negatives and therefore would not be recommended for the initial screening of CI. However, the specificity was relatively high (76.7%), so the model could be used for the secondary confirmation of CI. Prospective studies to evaluate the diagnostic model in predicting CI are warranted. In addition, our study was a single-center study and did not determine the causal relationship between microbiota dysbiosis and CI. Therefore, it is necessary to further validate with more longitudinal samples in multiple centers. Since the cause of CI or its clinical manifestations may not be necessarily addressed by the contribution of the gut microbiome as a whole, the individual species of the bacterial composition needs further delineation. Animal experiments illuminating the underlying mechanisms of specific gut bacterium in CI will be crucial in understanding the causal relationship.

This study characterized the dysbiosis in CI patient gut microbiota composition and function, specifically the decrease in BPB and increase in LAB abundances. We further explored the correlation between these alterations and multiple clinical parameters and developed a novel model that can be used for the secondary confirmation of CI diagnosis. To our knowledge, we are the first to demonstrate that the abundances and functions of LAB are significantly altered in CI patients. Our results shed light on the underlying mechanisms of CI, as well as indicated potential therapeutic targets for disease prevention and management.
